# Development of patient reported outcome measures assessing tumor pain intensity and tumor pain interference for individuals with neurofibromatosis type 1 and plexiform neurofibromas: qualitative findings

**DOI:** 10.1186/s41687-025-00877-2

**Published:** 2025-04-30

**Authors:** Nour Al Ghriwati, Kari Struemph, Staci Martin, Paige Little, Melissa Baker, Jason Levine, Cynthia MacKenzie, James Tonsgard, Elizabeth K. Schorry, Karin S. Walsh, Pamela L. Wolters

**Affiliations:** 1 Pediatric Oncology Branch, Center for Cancer Research, National Cancer Institute, National Institutes of Health, Bethesda, MD USA; 2 Clinical Research Directorate, Frederick National Laboratory for Cancer Research, Frederick, MD USA; 3 University of Kansas Medical Center, Kansas City, KS USA; 4 Nova Southeastern University, Fort Lauderdale, FL USA; 5 University of Chicago Pritzker School of Medicine, Chicago, IL USA; 6 Cincinnati Children’s Hospital and Medical Center, Cincinnati, OH USA; 7 Children’s National Hospital, The George Washington University School of Medicine, Washington, DC USA

**Keywords:** Pain assessment, Pain intensity, Pain interference, Qualitative, Neurofibromatosis type 1, Plexiform neurofibromas, Measure development, Patient-reported outcomes

## Abstract

**Background:**

Pain is a common symptom in individuals with neurofibromatosis type 1 (NF1) that often is associated with plexiform neurofibroma (pNF) tumors. To date, no patient-reported outcome measures have been validated specifically to assess pNF-related pain intensity or pain interference in this population. Such measures are sorely needed since pain is being considered as an outcome in clinical trials targeting reduction of pNF. The study aims were to: (1) obtain qualitative information from individuals with NF1 and pNFs about their pain and its measurement and (2) modify existing scales to assess pNF-related pain intensity and pain interference for NF1 clinical trials.

**Methods:**

For this multi-site, qualitative study, 56 individuals (26 children, 6–16 years; 30 adults, 18–68 years) with NF1 and pNF participated in a focus group and/or individual interview about pain intensity and pain interference (concept elicitation) and also provided feedback about existing pain measures (Numeric Rating Scale-11 and Pain Interference Index) assessing these domains (cognitive debriefing). Four additional waves of cognitive debriefing interviews further refined the measures. Qualitative concept elicitation data from transcripts were coded, analyzed using NVivo software, and thematic analysis was conducted using both deductive and inductive techniques. Additional themes and systematic problems and suggestions regarding the measures were gleaned from reviewing the field notes and interview transcriptions generated by the cognitive debriefing sessions.

**Results:**

Concept elicitation themes included descriptions of two types of pNF-related pain (chronic and episodic), variability of pain over time, varying ability to recall pain, lack of knowledge of pNFs, and the ways pain interferes with daily activities. Cognitive debriefing themes included information on how to rate pNF-related pain intensity apart from other pain; problems and suggestions regarding the measures included difficulty comprehending some items and preferences for alternative wording and formatting. Based on these qualitative results, the measures’ instructions, items, and formatting were modified to create the PAin INtensity Scale for plexiform neurofibromas (PAINS-pNF) and the Pain Interference Index for plexiform neurofibromas (PII-pNF) for administration on a mobile app or web-based platform.

**Conclusions:**

The PAINS-pNF and PII-pNF are promising self-report measures developed using patient engagement to evaluate tumor pain intensity and pain interference in NF1 clinical trials. The second phase of the study to provide reliability, validity, and normative data for individuals with NF1 and pNFs ages 8 years and older is underway.

**Supplementary Information:**

The online version contains supplementary material available at 10.1186/s41687-025-00877-2.

## Background

Neurofibromatosis 1 (NF1) is a rare genetic disease with multiple clinical manifestations, including plexiform neurofibromas (pNFs), which are benign tumors that grow along nerve sheaths. These tumors can cause pain and may significantly impact daily functioning and quality of life (QOL) [1, 2]. Pain has been reported in up to 65% of children with pNFs, ranging from mild to severe [3]. Even among children with pNFs taking pain medication, 93% reported pain that interfered with daily functioning at least somewhat [2]. From a large survey of adults with NF1, 55% reported tumor pain with 68% of these endorsing that it impacted their everyday life from a moderate to high degree [4]. In addition to pNFs, common sources of pain in NF1 are headaches, scoliosis, and other tumors [5], making the assessment of pNF-related pain difficult.

A qualitative study documented that pain is among the most frequent concerns for children through adults with pNFs [6]. The investigators also reported two distinct types of pNF-related pain: (1) a chronic, underlying pain usually experienced most days, which is relatively constant but seems to worsen through adulthood, and (2) an episodic pain, described as acute, localized pain near a pNF that is brief and occurs sporadically or from physical contact [7]. Participants further described how pNF-related pain interferes with daily life, such as limited participation in physical activities, missing school or social activities, and difficulty maintaining relationships [7]. Pain intensity and pain interference are separate but related constructs [8] both of which are important to understand in the context of NF1 and pNFs.

Valid pain assessment is imperative so that pain outcomes can be used as endpoints in clinical research trials targeting pNF reduction. In the absence of validated patient-reported outcome (PRO) measures of pain intensity and pain interference in this population, the Numeric Rating Scale-11 (NRS-11) [9] and the Pain Interference Index (PII) [8] were recommended by the Response Evaluation in Neurofibromatosis and Schwannomatosis (REiNS) International Collaboration for use in NF clinical trials assessing pain [10]. Furthermore, a clinical trial of selumetinib for children with NF1 and inoperable pNFs using these measures found significantly decreased tumor pain intensity and pain interference after one year of treatment [1]. Combined with reduced pNF volume, these PRO results contributed to the approval of selumetinib (Koselugo) in children with pNFs by the Food and Drug Administration (FDA) in April 2020. The FDA emphasizes the critical importance of PROs to evaluate clinical benefit (e.g., pain reduction, improved QOL) in addition to medical outcomes like tumor volume reduction for drug trials targeting pNFs. However, no valid PRO measures exist that specifically assess pNF-related pain intensity and its impact on daily functioning.

The current qualitative study followed FDA guidance to engage stakeholders in the development of PRO pain measures for NF1 clinical trials as part of the FDA Drug Development Tool program (#00061). The main aims of this study were: (1) to obtain qualitative information from adults, adolescents, and children with NF1, pNFs, and pain to understand their pain experience and how best to measure it, and (2) to use these data to modify existing PRO measures to assess pNF-related pain intensity (NRS-11) and pain interference (PII) in NF1 clinical trials.

## Methods

### Participants and settings

Individuals ages ≥5 years with documented NF1, with a pNF (at least 3 cm on longest diameter by physical exam or 2D MR imaging OR ≥ 3 mL by volumetric MR imaging), and who could understand and speak English were eligible. Participants also needed to report experiencing recent pNF-related pain rated *≥* 3 on the 0–10 NRS-11 pain intensity scale or report taking prescription pain medication and with recent pNF-related pain rated *≥* 1 on the NRS-11.

For the first wave of qualitative data collection, participants were recruited across 6 age bands (5–7, 8–11, 12–14, 15–19, 18–24, 25+) from four sites: National Cancer Institute (lead), University of Chicago Pritzker School of Medicine, Cincinnati Children’s Hospital and Medical Center, and Children’s National Hospital. Subsequent waves recruited individuals ages *≥* 8 years with Waves 3–5 completed at the National Cancer Institute due to COVID-19. The protocol (NCT02544022) was approved by each site’s Institutional Review Board. Age-appropriate informed consent and/or assent was obtained for all participants.

### Measures

*NRS-11.* The self-report NRS-11 has been used in numerous studies, including as a primary outcome measure, to assess pain intensity. This reliable and valid measure has been recommended for use in clinical trials by consensus groups and pain experts [11–13] and for individuals *≥* 8 years [14]. For this 0–10 scale (0 = no pain), the descriptions for a score of 10 vary widely across clinical practice and research studies [15]; thus, the investigators initially chose the commonly-used phrase “worst pain you can imagine.” They also generated initial pain questions based on clinical judgement and consultation with NF1 experts: Participants were asked to identify a pNF tumor and rate (1) the pain in that tumor at its worst, (2) their “overall tumor pain” at its worst, and (3) their “overall pain” at its worst – each over the past 7 days (Supplement 1).

*PII.* The PII is a 6-item questionnaire developed to assess the extent to which pain interferes with six different aspects of daily functioning on a 7-point Likert-type scale from 0 (not at all) to 6 (completely). The PII is a reliable, valid, feasible measure for chronically ill youth and adults, including those with NF1 [8] (Martin, personal communication, 2023). Participants completed either the child or adult self-report form (Supplement 2 and 3).

## Procedures

### Wave 1 concept elicitation groups/interviews and cognitive debriefing interviews

#### Focus groups and individual interviews

In either a focus group or individual interview, participants discussed NF1-related pain intensity and pain interference broadly (concept elicitation; CE) and provided specific feedback about the NRS-11 and PII (cognitive debriefing; CD). Both focus groups and individual interviews were conducted due to the rare nature of NF1 and scheduling constraints. Focus groups (planned for 2–8 participants) were age and sex matched. Interviews were conducted with children in the 5-7-year age group due to their developmental level.

Focus groups and interviews followed the same format, used parallel age-appropriate scripts, and were audio recorded. Two investigators co-led the groups, and one investigator conducted the interviews. Each group or interview started with CE where the moderators asked general open-ended questions to generate thoughts about pNF-related pain and how it affects daily life. Next, the moderators asked participants to complete the paper PRO measure assessing that domain. Once each measure was completed, the facilitators used a scripted CD process using the think-aloud method and verbal probing [16] and asked specific questions about the wording and other topics related to possible adaptations to these measures. Based on the Wave 1 CE and CD qualitative results, the investigators made changes to the two initial paper measures (NRS-11 v1.0 and PII v1.0) to create the first electronic versions using Scribe software administered on a tablet, named the PAin INtensity Scale-pNF (PAINS-pNF v2.0) and Pain Interference Index-pNF (PII-pNF v2.0).

### Waves 2 through 5 cognitive debriefing interviews

Waves 2 and 3 of CD interviews (CDIs) were conducted to further refine the measures based on qualitative results, detailed feedback from PRO development experts, and e-PRO guidelines [17, 18]. Wave 4 CDIs focused solely on the PAINS-pNF to assess a final minor modification made at the end of Wave 3 and to test possible delivery of both measures on a smartphone. After working with a digital health company, Metricwire, Inc. [19], to convert the measures to a mobile app to look like previously tested Scribe versions, a final Wave 5 of CDIs was conducted about how the measures worked on the smartphone app. The final adapted versions were the PAINS-pNF v6.0 and PII-pNF v5.0.

### Data analysis

#### Wave 1 qualitative concept elicitation (Thematic) analysis

All Wave 1 focus groups and interviews were professionally transcribed and deidentified. NVivo Qualitative Analysis Software, Version 11 was used to analyze the data through qualitative coding and queries. The three focus group moderators and a trained research assistant conducted the thematic analysis using combined deductive and inductive techniques [20]. An initial coding dictionary consisting of 25 codes was created a priori to guide measure development, which was updated as new codes were generated. Following each update, coders applied the new codes to previously coded transcripts. The first two transcripts were coded by all three moderators collaboratively to facilitate developing objective definitions for each code. Following these initial group sessions, remaining transcripts were divided among the moderators to code. Coding decisions were reviewed weekly, and consensus was reached before finalizing the codes in NVivo. The team then conducted a more in-depth qualitative analysis of all transcripts in which the codes were re-organized into themes and subthemes, resulting in our final coding dictionary that had 8 themes and 57 subthemes.

Theoretical saturation is defined as the point at which no new codes or themes are identified during continued review of the qualitative data [21]. The current study used an approach [22] whereby saturation was met after the first six focus groups and individual interviews revealed only 4% new information. Alternatively, with the threshold set at 0% new information [22], saturation was reached after the 12th focus group/interview.

### Waves 1 through 5 cognitive debriefing data: field notes and problem analysis

During CDI Waves 1–5, the investigators wrote detailed field notes [23, 24] and documented any difficulties or issues participants raised suggesting potential modifications to the measures. Then, the investigators reviewed the CD transcripts to identify problems that were systemic or potentially affecting the ability of individuals with NF1 to understand and complete the measures. They proposed solutions to each problem and adjusted the measures accordingly. These changes were evaluated in subsequent CDI waves.

## Results

### Participants

#### Wave 1 concept elicitation and cognitive debriefing focus groups and interviews

Eleven CE focus groups (*n* = 31 participants) were conducted with patients ages 8–62 years between 11/2015 and 11/2016 across all four sites. Individual CE interviews were completed with 26 patients, including 4 young children ages 6–7 years. However, one interview of a 15-year-old child was not recorded in error, making 25 evaluable patient interviews. Thus, the total sample included 56 participants, consisting of 30 adults and 26 children, ages 6–68 years. Since the first CD wave was conducted in the focus groups and interviews immediately following CE, participants in the Wave 1 CE/CD sessions are the same. Table 1 shows sample characteristics of Wave 1 participants. The four children < 8 years exhibited difficulties understanding the questions during the Wave 1 session; thus, these pain measures were not evaluated further in this age group.

**Table 1:** Sample characteristics of the wave 1 concept elicitation and cognitive debriefing focus groups and individual interviews

	Total sample	Adults	Children
Total N^a^	56	30	26^b, c^
Age (years)			
Mean age (SD)	25.3 (16.5)	36.6 (14.8)	12.1 (3.5)
Range	6–68	18–68	6–16
Education (years)^d^			
Mean	13.9 (2.1)	13.2 (1.5)	14.7 (2.4)
Range	10–19	11–16	10–19
Sex			
Male	23 (41.1%)	14 (46.7%)	9 (34.6%)
Female	33 (58.9%)	16 (53.3%)	17 (65.4%)
Race			
Caucasian	35 (62.5%)	17 (56.7%)	18 (69.2%)
African American	12 (21.4%)	8 (26.7%)	4 (15.4%)
Latino	2 (3.6%)	2 (6.7%)	
Multiple	6 (10.7%)	2 (6.7%)	4 (15.4%)
Missing/Did not report	1 (1.8%)	1 (3.3%)	
Received special education	38 (67.9%)	19 (63.3%)	19 (73.1%)
Psychiatric diagnosis	30 (53.6%)	17 (56.7%)	13 (50%)
Learning disability	28 (50%)	15 (50%)	13 (50%)
Pain medication	39 (69.6%)	24 (80%)	15 (57.7%)
Disease severity			
Mild	12 (21.4%)	7 (23.3%)	5 (19.2%)
Moderate	33 (58.9%)	15 (50%)	18 (69.2%)
Severe	10 (17.9%)	7 (23.3%)	3 (11.5%)
Missing/Did not report	1 (1.8%)	1 (3.3%)	

^a^Focus group interviews ranged from 58 to 120 min. Individual wave 1 interviews ranged from 19 to 87 min

^b^27 children participated in a focus group or interview; however, an audio file from one child’s interview (15-year-old female) was not recorded in error, so the data from this participant could not be formally analyzed

^c^Number of children within the following predetermined pediatric age groups: four 5–7 years, six 8–11 years, five 12–14 years, and twelve 15–19 years in high school

^d^Years of education for adults or years of parental education for child participants

### Waves 2–5 cognitive debriefing interviews

After the initial modifications to the measures based on Wave 1 qualitative groups and interviews, subsequent CDI waves were conducted across several age groups *≥* 8 years to evaluate the changes further. Table 2 presents the participant characteristics from CD waves 2–5.

**Table 2:** Sample characteristics of the waves 2–5 cognitive debriefing interviews

WAVE	2	3	4	5
Dates	2/2018-2/2019	6/2020-12/2020	5/2021-7/2021	8/2022-9/2022
Interview length (min)	16–29	21–46	9–20	7–19
Total N	27^a^	11^b^	8^c^	6^d^
< 18^e^	15	4	3	2
≥ 18	12	7	5	4
Age (years)				
Mean age (SD)	22.48 (16.22)	25.00 (13.62)	24.63 (14.00)	29.17 (14.36)
Range	8–60	9–45	11–46	15–48
Education (years)^f^				
Mean	14.67 (2.66)	14.91 (2.07)	15.13 (1.81)	15 (2.53)
Range	11–22	12–18	12–18	11–18
Sex				
Male	17 (63.0%)	7 (63.6%)	5 (62.5%)	4 (66.7%)
Female	10 (37.0%)	4 (36.4%)	3 (37.5%)	2 (33.3%)
Race				
Caucasian	20 (74.1%)	8 (72.7%)	6 (75.0%)	6 (100%)
African American	3 (11.1%)	1 (9.1%)	1 (12.5%)	0 (0%)
Latino	1 (3.7%)	0 (0%)	0 (0%)	0 (0%)
Asian	1 (3.7%)	1 (9.1%)	1 (12.5%)	0 (0%)
Other	2 (7.4%)	1 (9.1%)	0 (0%)	0 (0%)
Received special education	15 (55.6%)	4 (36.4%)	5 (62.5%)	2 (33.3%)
Psychiatric diagnosis	5 (18.5%)	4 (36.4%)	3 (37.5%)	2 (33.3%)
Learning disability	11 (40.7%)	3 (27.3%)	1 (12.5%)	2 (33.3%)
Pain medication	14 (51.9%)	7 (63.6%)	4 (50.0%)	NC^g^
Disease severity				
Mild	5 (18.5%)	3 (27.3%)	3 (37.5%)	NC^g^
Moderate	16 (59.3%)	4 (36.4%)	3 (37.5%)	NC^g^
Severe	5 (18.5%)	4 (36.4%)	2 (25.0%)	NC^g^
Missing/Did not report	1 (3.7%)	0 (0%)	0 (0%)	0 (0%)

^a^For Wave 2, all participants were new patients who had not taken part in Wave 1

^b^For Wave 3, five out of the 12 patients (41.7%) had participated in Wave 1 or 2. While 12 participants completed the interview, one interview was not recorded in error so it could not be transcribed to use for problem coding

^c^ For Wave 4, four out of the eight participants (50%) had participated in Wave 2 or 3

^d^ For Wave 5, six out of the six participants (100%) had participated in Wave 1, 2, 3, or 4

^e^ Breakdown of < 18 (including 19 years old still in high school) in Wave 2: nine 8–12 years, six 13–19 years; Wave 3: two 8–12 years, two 13–19 years; Wave 4: one 8–12 years, three 13–19 years; Wave 5 (only about the smartphone app): no 8–12 years, two 13–19 years

^f^ Years of education for adults or years of parental education for child participants

^g^ Not collected

### Pain intensity results

#### Wave 1 CE themes

Five broad themes emerged from the Wave 1 CE qualitative data that related to the development of a measure to assess pain intensity in individuals with NF1 and pNFs as presented below; supporting quotes are listed in Table 3.

**Table 3:** Wave 1 concept elicitation and cognitive debriefing themes supporting quotes for pain intensity

CE themes	Subthemes	Supporting quotes
Different types of pNF-related pain	Chronic	“Constantly there” “It’s going to always hurt” “Pain is every single day”
	Episodic	“A shock pain” “Like an electricity pain, it will come really fast, and then it leaves” “If I hit against something”
	Both types	“If I use my arm a lot it feels achy, but like if I was to like hit my arm or something it would feel completely different, like a very sharp pain” “I think the narrowing definitions […] to find more precise areas of the kinds of pain helps […] to clarify what it is I’m actually answering”
Pain variability over time	Fluctuation within a day	“If I feel pain in the morning, I know it’s going to be bad by the end of the day” “Usually, evenings are worse than the rest of the day”
	Fluctuation within a week	“Good days and bad days” “One day might be a 5 and the next day might be a 9” “Some days it can be worse than others and it can get worse and get better” “Well, they like say the past week but it’s usually not just one number the past week. It’s up and down”
Varying ability to recall pain	Able to remember pain	*Daily recall*: “Today” “I don’t know, except for today”
		*Weekly recall*: “The week … because a month can be too old” “For the past seven days I think I could be pretty accurate”
		*Recall of pain when linked to specific events*: “I take the tramadol three times a day and sometimes four times. Yesterday was four times a day…Yesterday was a real bad day for me” “I just had a plexiform removed out of my neck November 17th ”
	Not able to remember pain	*When asked about remembering their pain*: “My memory sucks” “I’m not sure I could say specifically”
	Difficulty understanding the phrase “the past 7 days” in general	When asked to define the past 7 days: “Like the past couple of days”
Lack of knowledge of pNF tumors		“I don’t know if I have [a pNF tumor] or not” When asked about their most important tumor pain, a child responded: “My most important pain is my leg pain,” but parent noted no pNF tumor in that area.
Current pain assessment techniques	Various types of pain measurements used	*Different types of verbal pain ratings*: “My doctors always just ask me when I go there, what’s your pain at this very moment and what’s your average pain” “Here they ask me in the past week, and I guess like sometimes at Children’s [National Hospital] they ask I guess in the [past] month”
		*Different types of numeric rating scales*: When asked what doctors use to measure pain: “On a scale from one to 10” “I think some doctors use the faces, like the smiley faces, a happy face”
		*Open Ended Verbal Descriptors*: When asked for other ways to rate their pain: “They really just talk to me. They usually just do what you’re doing, asking me how my pain is, how my back is”
		*Other Measurement Types*: “Keep a big chart […] where I rate the pain, what kind of pain it was, and when it was for my doctors – like what time of the day” “I started really tracking it myself. I could probably find calendars that I have, like day planners and stuff”
	Mixed attitudes about current pain measurements	*Dislikes*: When discussing the crying face: “Because I’ve been dealing with it for too long and I’ve cried all my tears; it becomes part of your daily life when it hurts you” When discussing the 0–10 scale: “what I consider a 10 is different from what someone else considers a 10” “There’s no substance to it, there’s no story to it” “I think they should ask did it stop you from your daily abilities” “I don’t think they are too accurate” When discussing frequency of assessments: “Well, they like say the past week but it’s usually not just one number the past week. It’s up and down”
		*Likes*: When discussing the 0–10 scale: “It kind of works because like some days it can be worse than others and it can get worse and get better” “I think it’s pretty accurate because you know pain is usually on a scale of one to 10” “I think it is a good way” “The way it’s been done for ages” “It is like you are used to it by now”
Pain differentiation	Able to differentiate	*One source of tumor pain*: “There really aren’t others to rate”
		*Tumor specific location*: “I think pretty well because it asked for a specific body – which part hurts. I am able to identify what part of my body it is and then describe that pain for that one” “It is pretty easy to say the one in my shoulder is causing me pain or the one in my neck […] It is almost a no-brainer”
		*Nature of NF Pain*: “My NF pain is different than my arthritis pain” “Each kind of have their own pain – different kinds of pain” “Because your head pain is not the same as your leg pain” “If it’s from the neurofibromatosis, it’s higher intensified pain”
	Some difficulty differentiating	*Close tumors*: “I had like two tumors in my lower back and I couldn’t tell which one it was because they were close together” When asked how easy it was to differentiate two close tumors: “Sometimes you can sometimes you can’t” “I can’t pinpoint one specific because I have too many to say one specific”
		*Similar pain*: When asked if the pain is related to migraines or the plexiform on their head: “It is like both”
		*Non-NF pain*: When asked if their pain was from NF or something else: “Stress or not enough sleep or eating properly” “I know I hurt when it rains, but if it is arthritis, I don’t know. It could very well be, but I don’t know”
	Not able differentiate	“I couldn’t really say when it’s a tumor pain or when it’s just a regular pain”
Difficulty selecting pNF tumor location	Pain from multiple tumors	When asked to choose their most important tumor: “I kind of put a couple different ones because it’s hard to pick one specific” “Well, I’ve got a couple of tumors that really bother me”
	Internal tumor source	“I don’t think any of my ones that you can see hurt, it’s all the ones inside that hurt”
	Tumor location or pain source unknown	“I don’t know which one this is, if it’s a plexiform or not … I don’t know what they are, I never asked, it just hurts”
	Pain not contained to tumor location	“My specific pain isn’t a specific tumor, it’s the nerve that’s being affected by the tumor”
Documenting a pNF tumor location		*Figure preference*: When asked about their preferences for how to indicate the location of the pNF they are rating: “If you had the picture of the body like they do of the front and the back and the side…” “The drawing might actually be better because I can pretty accurately trace where the tumor goes”
		*Write in preference*: Writing the location would be difficult: “for you to ask me to spell anything out, I’m not going to do it” “I can’t write that so I’m not going to be able to tell you”
		*Figure and write in preference*: “I say a little bit of both where you can write it down and circle it on the picture where the exact spot would be”
		*Difficulty with figure*: “I get confused on the right or the left on those things” “I think if they are going to do the pictures, they need to specifically show the right side, the left side […] definitely have it labeled”

##### CE theme 1: Different types of pNF-related pain

Participants reported various types of pain that were unrelated to their pNF tumors, including migraines, arthritis, abdominal pain, and skin irritation, and they used different adjectives to describe their pain. However, clear subthemes emerged indicating that they experienced two different types of pNF-related pain: chronic and episodic.

*Chronic and episodic pain.* Many participants described an underlying chronic pain that is usually present and a different episodic pain that is sudden and shorter in duration, which occurs unpredictably or when the tumor is bumped. Some people experience both types of pNF-related pain and some experience just one type. One participant specifically stated it was helpful to have these more specific pNF-related pain types to rate.

##### CE theme 2: Pain variability over time

Many patients reported that their chronic pNF-related pain ebbs and flows, both within a day, over several days within a week, or over longer periods. Many patients with pNFs noted that activity regularly exacerbated their pain and pain got worse throughout the day.

##### CE theme 3: Varying ability to recall pain

Participants’ discussions indicated varying ability to recall pain depending on the timeframe and situation that included three main subthemes as described below.

*Able to remember pain*. This first subtheme included reports of how long participants could remember their pain and what helped them to remember it. To rate their pain intensity accurately, several participants only said for “today” while others felt that a week would be a reasonable recall period. Additionally, some participants remarked that they often linked their pain to specific events, such as how much medicine they took or a medical procedure, which helped their recall.

*Not able to remember pain.* The second subtheme reflected that patients are “not able to remember” their pain. Many participants discussed difficulty recalling specific information about past pain experiences due to poor memory.

*Difficulty understanding the phrase “the past 7 days.”* The third subtheme came from some participants having difficulty understanding “the past 7 days.” When asked about what this phrase means, some participants were unable to define it correctly.

##### CE theme 4: Lack of knowledge of their pNF tumors

This theme is based on participants who reported pain but were unsure about whether they had a tumor or they did not have a pNF in the area of their reported tumor pain.

##### CE theme 5: Current pain measurement techniques

When discussing current pain measurement techniques being used in NF1 clinics, participants indicated that medical providers use inconsistent approaches to ask about pain, and patients have mixed attitudes about these pain measurements. These two subthemes are described below.

*Various types of pain measurements used by medical teams.* Participants indicated that there are many ways they are asked to rate their pain at clinic visits, including average pain, current pain, worst pain, overall pain, and pain frequency. Participants also noted that rating current or average pain does not accurately reflect their pain because it often is variable. Most participants reported that they are asked to rate their pain on 0–10 scales, either with or without faces or pictures; however, at least one participant said that they dislike choosing the crying face for chronic pain because it is part of their daily life. Additionally, the recall period on the 0–10 scale that doctors used varied widely (e.g., currently, daily, over the past week, the past month). Medical providers also asked them to describe their pain verbally. Some participants suggested a good way to measure their pain would be to keep a chart or diary.

*Mixed attitudes about current pain measurement using the NRS-11.* Participants described both negative and positive attitudes about using the NRS-11 for rating their pain. The most frequent reason for disliking the 0–10 scale was that patients had different interpretations for the level of pain that each number represented (i.e., a given number might mean different levels of pain to different people). However, this reason would not negatively affect the use of the NRS-11 in clinical trials because the focus is on evaluating changes in pain over time within subjects and not on differences in levels of pain between subjects. Some also felt that the numeric scale did not capture important aspects of NF1 pain, such as how it affected their day-to-day life. Finally, some participants expressed difficulty summarizing their pain using one number because of the variability. They often expressed dissatisfaction with the typical weekly recall period of the NRS-11 due to frequent fluctuations in their pain, indicating that one rating over the past week would be inadequate to describe their pain intensity. However, others expressed positive attitudes about the NRS-11 because they felt that it was accurate and/or the best way they could communicate their pain intensity to the medical team and the scale was familiar to them.

##### Wave 1 CD themes

During the CD portion of the Wave 1 focus groups and interviews in which participants completed and provided feedback on the NRS-11, three additional themes emerged about this scale as described below (see Table 3 for supporting quotes).

*Ability to differentiate pNF pain.* Many patients can differentiate between various types of pain experienced, such as pain from their pNFs versus other complications and between pNF-related pain in different areas of their body. Participants who expressed some difficulty differentiating between pains noted that it was because of not knowing where their pNFs were located or which pNF they should focus on. Others said that they had many small tumors close together making it difficult to identify which one was the source of the pain. A few said it would be hard to distinguish tumor pain from other pains. However, most participants said that they would be able to rate the pain intensity of a target pNF location identified by the medical team.

*Difficulty selecting a pNF location.* Many participants expressed difficulties choosing a tumor pain location to rate because they had multiple pNF tumors or were unsure which tumors were pNFs versus other tumors or of their specific tumor locations. For these reasons, patients preferred having the medical team select the target pNF location to rate rather than self-selecting a location.

*Documenting pNF location.* Participants were asked their preferences for how to indicate the pNF location they are rating. Most preferred to identify their tumor location on a human figure drawing as they found writing the location difficult. However, some preferred to write the location or have both options while others expressed that they would have difficulty indicating their tumor location on the figure independently (e.g., figure is not exact to human body, getting right/left confused). Therefore, the measure was modified to have a medical provider circle the pNF tumor area on the human figure drawing with a R on the right side and L on the left side, which is shown to the participant when answering the questions. Slightly different versions of the figure were explored in subsequent CDI waves.

### Wave 1–5 CD systematic problems and suggestions

When analyzing the data across all five CD waves, problems in the items, scale, or format were identified based on participants’ comments about the NRS-11 for assessing their pNF-related pain that led to changes in the measure. For example, many patients were confused by the questions that asked them to rate the intensity of their overall pain and overall tumor pain on the modified NRS-11, so those items were deleted. Some children demonstrated difficulty understanding the two types of pain without explanation as they may not have experienced both; thus, descriptions and a training session were added. Many patients also expressed concerns about the using “worst pain you can imagine” as the verbal descriptor for the 10 on the scale and suggested wording it differently. Some reported preferring daily pain ratings instead of over the past week due to difficulty remembering their exact pain or variability in their level of pain.

### Pain interference results

#### Wave 1 CE themes

During the Wave 1 CE focus groups and interviews, three themes were evident when talking with patients about how pNF-related pain interfered with daily life that were related to modifying the PII for individuals with NF1 and pNFs. Supporting quotes for these themes are in Table 4.

**Table 4:** Wave 1 concept elicitation and cognitive debriefing themes supporting quotes for pain interference

CE themes	Supporting quotes
Physical Interference	“I can’t turn my neck to talk to you. … Because it really hurts.” “I can’t wash my hair. I can’t brush or comb my hair” “I can barely run anymore” “I’ve got to get up and move because the sitting is…painful”
Physiological Interference	“I will lose my focus because I am just kind of sitting there rubbing my legs…” “I am always tired…most of the time, I feel like I just want to lie down and go to sleep” “I got so bad in pain. … I couldn’t eat nothing, I did not have an appetite.”
Social-emotional Interference	“I had no patience for my kids” “You don’t feel like getting out there and being all bubbly” “I get a little aggravated, maybe a little sad sometimes” “With me, it’s not an everyday thing unless I touch somebody, and they’ll touch a fibroma the wrong way”

*Physical interference.* Regarding physical manifestations, respondents talked about having limitations while engaging in their usual activities. Even sitting for long periods of time may cause an increase in pNF-related pain. Focus group members referred to daily self-care tasks that are more difficult because of pain, such as showering and combing their hair. Among more challenging physical activities, people shared pain-related limitations surrounding hiking, running, and engaging in contact sports.

*Physiological interference.* With respect to the physiological impact of pNF-related pain, patients described cognitive difficulties such as paying attention. Other reported physiological effects of pain included poor appetite, lack of energy, and trouble sleeping.

*Social-emotional interference.* The pain from pNF tumors had an emotional impact on patients, as demonstrated by comments about irritability and sadness. Participants also described pain causing difficulties being physically close to others, since their tumor area may get touched or bumped. Tumor pain also interfered with fun social activities.

### Wave 1–5 CD systematic problems and suggestions

An analysis of the CD data pointed to several problems based on the participants’ comments about the PII for assessing their pNF-related pain interference as described below. Additionally, the researchers combined the child and adult self-report scales into one measure that can be used across all ages due to the wide age range often enrolled in NF1 clinical trials.

*Suggestions for additional items.* Participants noted aspects of pNF-related pain interference that were not covered by the original six items. Specific domains they suggested including were sitting or standing for long periods of time, sleeping, appetite, and intimacy or physical closeness.

*Comprehension difficulties with certain terminology.* A few participants did not understand some of the words used in the PII or had difficulty with pronunciation of some words. Importantly, people expressed differing opinions about what constituted a leisure activity, ranging from sports to resting.

### New pain measures

Based on the qualitative findings, both measures were modified to be administered electronically using a secure mobile app or web-based platform for individuals with NF1 and pNFs ages *≥* 8 years. The PAINS-pNF (Table 5) and PII-pNF (Table 6) item tracking matrix shows a detailed breakdown of main changes and supporting data. A text-to-speech option is available for individuals with reading difficulties. Descriptions of the two pain types and instructions about the measures are reviewed in a manualized training session to be completed prior to administration. The final versions of the measures are described below.

**Table 5:** Changes to the NRS-11 based on qualitative CE and CD data

PRO concept	Initial NRS-11 concept	Changes made and rationale	Supporting CE/CD themes and identified problems from CD	Final PAINS-pNF concept
Types of pNF-related pain rated	• 3 items assessing: o Overall pain o Overall tumor pain o pNF tumor-specific pain	• Deleted overall pain items due to confusion • Developed 2 new pain intensity items based on different types of pNF-related pain	• 2 types of pNF tumor pain: chronic and episodic pain • Confusion about rating overall pain items • Pain differentiation between various types of pain and pNF tumor pain in different parts of the body	• 2 items assessing: o Spikes of tumor pain (episodic) o Usual (chronic) tumor pain • Added descriptions of pain types
Recall period	• Items assessed pain over past seven days	• Changed to daily rating of pain intensity	• Remember pain better within the past day • Variability in pain ratings in the past day/week • Inconsistent recall periods for NRS-11 scales • Not understanding the phrase “the past 24 hours” when asked	• 24-hour recall completed from “the time you went to bed last night until now (including overnight)”
Frequency and timing of ratings	• One-time retrospective measure	• Changed to daily administration across 1–2 weeks before medical visits	• Willingness to complete measure daily as part of a clinical trial • Increase in pain throughout the day associated with activities • Fluctuation of pain across days	• Measure administered every evening (at a patient-selected time) for 1–2 weeks before medical visit
Mode of administration	• Paper-and-pencil measure	• Changed from paper to electronic administration via mobile app or web-based platform	• Some preference for use of mobile app when asked • Difficulty with writing using paper and pencil • Electronic administration ease of use • Suggestions for reminder notifications	• Administered electronically via Metricwire mobile application
Scale anchors	• Scale anchors ranging from “no pain” to “worst pain you can imagine”	• Modified scale anchors • Added type of tumor pain to the scale anchors to remind participants what tumor pain is assessed in each question	• Dislike of the phrase “worst pain you can imagine” for the #10 descriptor on the 0–10 scale • Difficulty remembering the type of tumor pain being rated in each question	• Scale ranges from 0="no tumor pain” to 10="worst tumor pain possible (spikes)” or “worst tumor pain possible (chronic/usual)”
Tumor location selection	• Participants selected their own pNF tumor pain location and rated the pain for that tumor	• Changed from asking participants to self-select a pNF tumor to having the medical team select a target pNF tumor pain location for them to rate	• Difficulty self-selecting a tumor pain to rate, especially if there are multiple tumors • Preference for having the medical team select a target tumor to rate	• The medical team chooses a tumor location with patient input for the patient to their rate pain
Documenting pNF tumor location	• Participants indicated via writing on the paper form their selected tumor pain	• Changed pNF location to be documented and referenced via circling it on human figure drawing	• Preference to identify and circle tumor location on a human figure drawing • Circling area on human figure to show tumor location easier for patients to understand • Difficulty specifying tumor location via writing • Some patients not sure about whether they have a PN tumor or where it is located	• A medical provider circles the pNF tumor area on the human figure drawing with a R on the right side and L on the left side for patients
Voiceover feature	• Paper-and-pencil measure did not have voiceover feature	• Added a text-to-speech voiceover feature	• Difficulty reading words • Documented learning disabilities with impairment in reading for many patients with NF1 • Participants responded “yes” when asked if they wanted a voiceover feature button to read aloud instructions and items	• Voiceover feature included to read questions aloud to patients if needed

**Table 6:** Changes to the PII-pNF based on qualitative CE and CD data

PRO concept	Initial PII concept	Changes made and rationale	Supporting CE/CD themes and identified problems from CD	Final PII-pNF concept
Type of pain assessed	• Measure assessed overall pain interference	• Added “tumor pain” to each question stem to clarify tumor pain interference • Added instructions for participants to think about their plexiform neurofibroma tumors	• Patients reported pain from multiple sources and needed to focus the ratings on pNF-related tumor pain	• Measure assesses “tumor pain interference”
Universal measure across age groups	• Separate child and adult versions of the measure	• Consolidated child and adult versions into one scale for all ages 8 + years	• Experts desire to have same measure across all age groups in clinical trials • Needed to have one measure that is appropriate for all ages for use in clinical trials	• PII-pNF v5.0 is for patients ages 8 + years
Item number, scales, & order	• Six items assessing pain interference	• Added & modified items to reflect participants’ experiences within physical, physiological, & social/emotional domains	• Participants felt that there were aspects of pNF-related pain interference that were not covered by the original six items • Described additional types of pain interference that fell within physical, physiological, and social/emotional domains	• Twelve items grouped within the domains that they assess
Item terminology	• Items assessed pain interference with leisure activities or mood	• Simplified item terminology to be more easily understood by children & adults	• Difficulty understanding some of the words used in the PII, such as “leisure,” and “mood” • Suggestion that the measure use the word “feelings” instead of “mood”	• Replaced “leisure activities” with “things you enjoy” and “mood” with “feelings”
Examples within question items	• Items broad and with few examples to explain each interference concept	• Added examples of activities within most items to increase clarity	• Statements suggesting that people may have differing opinions about what constitutes a leisure activity, usual physical activities, challenging physical activities, or work outside of the home	• Items include examples for increased clarity as to the activities included within each item
Mode of administration	• Paper-and-pencil measure	• Changed from paper to electronic administration via mobile phone application	• Preference for use of mobile app • Difficulty writing using pencil and paper • Electronic administration ease of use • Suggestions for reminder notifications	• Administered electronically via Metricwire mobile application

*PAINS-pNF 6.0*. The final version of the PAINS-pNF is a 2-item electronic self-report questionnaire to assess pNF-related pain intensity. Respondents are asked to rate the pain intensity of a target tumor area of the body (tumor pain intensity), selected by the medical team with patient input, which is circled in red on a human figure drawing. The first item asks patients to rate their spikes of tumor pain (episodic) in this area and the second item asks them to rate their usual (chronic) tumor pain in this area, both on a scale from 0 (no pain) to 10 (worst pain possible) from the time you went to bed last night until now. When completing the questionnaire at home in the evening (at a patient-selected time), this wording provides an approximately 24-hour period that is understandable for all ages. The goal is for patients to complete the measure every evening for 7 days (with a minimum of 3 days) prior to each appointment for a clinical trial, with ratings averaged for each item over the 7 days and a total score calculated by adding the two item averages.

*PII-pNF 5.0*. The final version of the PII-pNF is a 12-item electronic self-report questionnaire assessing pNF-related pain interference in daily life. All items ask respondents to consider the pain related to their pNF tumors in the past 7 days. Specifically, items ask how much their tumor pain has made it hard for them to engage in various physical activities (e.g., usual activities, challenging physical activities, day-to-day tasks) and how much it has impacted physiological factors (e.g., attention, sleep), and their social-emotional functioning (e.g., mood, relationships). Patients respond on a 7-point Likert-type scale with 0 = not at all, 3 = some, and 6 = completely. The goal is for patients to complete the measure one evening within 1–2 days prior to their clinical trial appointment. The total score is the mean of the 12 items.

## Discussion

The PAINS-pNF and PII-pNF are the first PRO measures that specifically assess pNF-related pain intensity and pain interference in individuals 8 years and older with NF1. The development of these new measures was an iterative process that progressed through several stages of qualitative data collection from concept elicitation to cognitive debriefing. As each modification was based on patient and expert feedback regarding the use of these measures, the tools have excellent face and content validity for evaluating changes in pNF-related pain for NF1 clinical trials. In addition, these data were obtained following rigorous qualitative methods consistent with FDA requirements of PRO development [25] and best practices of electronic administration [26].

The PAINS-pNF assesses the severity of two kinds of pNF-related pain, chronic and episodic, which were described by many participants in the current research and reported in another qualitative study of patients with NF1 and pNF [7]. The development of the PAINS-pNF aims to fulfill the FDA’s preference for PRO measures that assess symptoms proximal to the disease for clinical trial outcomes versus domains that can be impacted by other aspects of patients’ lives [25, 27]. The PII-pNF assesses pNF-related pain interference in a range of daily activities as described by individuals with NF1 and pNF. Participants said that this measure told the “story” of how their pNF-related pain affected them every day, which they reported as a critical aspect to convey to their medical providers and important to assess in addition to the severity of their pain. Both measures were adapted for electronic administration using a mobile app or web-based platform to allow remote pain assessment in real time in the home environment that will improve ecological validity [28] and data accuracy [28].

These measures fill the critical need for PROs that specifically assess aspects of pain related to pNF tumors that are not available from other scales (e.g., PROMIS Pain Interference). It is recommended that both measures be administered together in clinical trials to assess these aspects of tumor pain longitudinally and their relationship to each other. Although developed specifically for NF1 research, these new measures also could be used clinically to monitor changes in pain.

### Limitations

Research regarding focus groups for qualitative inquiry suggests conducting 3–5 groups per segment (i.e., an identified group of people) and having between 6 and 10 participants per group. NF1 is a rare disease, which inherently limits recruitment. When adding the additional criteria of patients needing to have a pNF and experience pain, this study was unable to meet that gold standard. However, there is acknowledgement among experts that group size and number may need to be modified depending on the complexity of the topic (greater complexity: fewer group members) and the size of the population (smaller population: fewer groups) [29, 30]. For the current study, scheduling larger focus groups was difficult, particularly with children, so smaller groups and individual interviews were completed. In cases of limited samples, a combination of focus groups and individual interviews is acceptable and may help obtain different types of data with a wider breadth of information coming from focus groups and more depth of information coming from individual interviews [31].

Balanced recruitment between adult and child/adolescent age bands was not possible given that children with NF1 have less chronic pain [7], lower pain frequency, and fewer pain sources than adults [5], and ongoing clinical trials resulted in decreasing pain in children and adolescents with pNFs [1]. However, results did not suggest substantial differences between the content of qualitative responses provided by younger and older participants; rather, comprehension of the measures was the main difference. Thus, the measures were developed to be understood by children *≥* 8 years of age, and the data collected in the quantitative phase will evaluate the reliability and validity of the measures across age groups and determine the lowest age of administration.

### Future research

The psychometric properties of these two new PRO pain measures currently are being evaluated in the quantitative phase of this study. Results will determine their suitability for use as outcome measures to assess pNF-related pain intensity and pain interference in NF1 clinical trials, either as key secondary outcomes in conjunction with volumetric MRI to evaluate pNF reduction or as primary outcomes to assess changes in pNF-related pain related to analgesic medications.

## Conclusions

Using qualitative data from patients and expert input, two PRO measures were developed that assess pNF-related pain intensity and pain interference in individuals with NF1 ages 8 years and older. These measures are to be completed electronically from home prior to clinical trial endpoint evaluations. It is critically important to have PROs with excellent content validity to assess clinical benefit in trials targeting the reduction of pNFs and pain in NF1 across the lifespan. Validation of these tools is underway, and they will be available for use after publication of the quantitative results.

## Electronic supplementary material

Below is the link to the electronic supplementary material.

Supplementary Material 1

Supplementary Material 2

Supplementary Material 3

## Data Availability

The data that support the findings of this study are available from the corresponding author upon reasonable request.

## Abbreviations

CE: Concept elicitation
CD: Cognitive debriefing
CDI: Cognitive debriefing interviews
FDA: Food and drug administration
NF1: Neurofibromatosis type 1
NRS-11: Numeric rating scale-11
PAINS-pNF: PAin INtensity scale for plexiform neurofibromas
PII: Pain interference index
PII-pNF: Pain interference index for plexiform neurofibromas
pNF: Plexiform neurofibroma
QOL: Quality of life
REiNS: Response evaluation in neurofibromatosis and schwannomatosis
