# Supplementary material for: Development of patient reported outcome measures assessing tumor pain intensity and tumor pain interference for individuals with neurofibromatosis type 1 and plexiform neurofibromas: qualitative findings
Source: J Patient Rep Outcomes. 2025 Apr 30;9:46. doi: 10.1186/s41687-025-00877-2 (PMC12044096; doi:10.1186/s41687-025-00877-2)
Supplement: Supplementary file 1 — Supplementary Material 1 [file 41687_2025_877_MOESM1_ESM.pdf]

Participant's Study ID:______________
Protocol:___________________________
Site:___________ Date:____________

Pain Questionnaire
(Self-report Form)

Numeric Rating Scale – Pain Intensity

Below are some questions about how much you hurt. The first two questions are about how much your plexiform neurofibroma tumor or tumors hurt, and the other question is about how much you hurt from all types of pain.

Below are lines with numbers from 0 to 10 where 0 means no pain and 10 means the worst pain you can imagine.

1. We would like you to pick one tumor and tell us how much that one tumor hurts at each study visit.

Where on your body is that tumor?____.

Please circle the one number that best describes the pain in that one tumor at its **worst** during the past 7 days.

0 1 2 3 4 5 6 7 8 9 10
No pain — Worst pain you can imagine

2. Please circle the one number that best describes your overall tumor pain at its **worst** during the past 7 days.

0 1 2 3 4 5 6 7 8 9 10
No pain — Worst pain you can imagine

3. Please circle the one number that best describes your overall pain at its **worst** during the past 7 days.

0 1 2 3 4 5 6 7 8 9 10
No pain — Worst pain you can imagine

Adapted by [blinded] (8/23/13)
