# Supplementary material for: Development of patient reported outcome measures assessing tumor pain intensity and tumor pain interference for individuals with neurofibromatosis type 1 and plexiform neurofibromas: qualitative findings
Source: J Patient Rep Outcomes. 2025 Apr 30;9:46. doi: 10.1186/s41687-025-00877-2 (PMC12044096; doi:10.1186/s41687-025-00877-2)
Supplement: Supplementary file 2 — Supplementary Material 2 [file 41687_2025_877_MOESM2_ESM.pdf]

Participant's Study ID:______________
Protocol:___________________________
Course Number:____________________
Date:______________________________

Child/Adolescent Pain Questionnaire

(Self-report form for ages 6 – 18 years)

Pain Interference Index – Pain Interference

Below you will find a list of questions about you and your pain. Please answer each question by circling a number between 0 and 6.

We are asking about your pain during the past 7 days.

In the past 7 days

Has your pain:		Not at all			Some			Completely
**1.**	made it difficult for you to do schoolwork?	0	1	2	3	4	5	6
**2.**	made it difficult for you to do activities outside school (leisure activities)?	0	1	2	3	4	5	6
**3.**	made it difficult for you to spend time with friends?	0	1	2	3	4	5	6
**4.**	affected your mood?	0	1	2	3	4	5	6
**5.**	affected your ability to do physical activities (like run, walk up stairs, play sports)?	0	1	2	3	4	5	6
**6.**	affected your sleep?	0	1	2	3	4	5	6

Adapted by the Neurobehavioral Group, NCI (8/23/13)
