# Supplementary material for: Development of patient reported outcome measures assessing tumor pain intensity and tumor pain interference for individuals with neurofibromatosis type 1 and plexiform neurofibromas: qualitative findings
Source: J Patient Rep Outcomes. 2025 Apr 30;9:46. doi: 10.1186/s41687-025-00877-2 (PMC12044096; doi:10.1186/s41687-025-00877-2)
Supplement: Supplementary file 3 — Supplementary Material 3 [file 41687_2025_877_MOESM3_ESM.pdf]

Participant's Study ID:______________
Protocol:___________________________
Course Number:____________________
Date:_______________________________

Adult Pain Questionnaire

(Self report form for 18+ years)

Pain Interference Index – Adult

Below you will find a list of questions about you and your pain. Please answer each question by circling a number between 0 and 6.

Please note that we are asking about your pain during the past 7 days.

	**Has your pain:**	Not at all			Some			Comp-letely
1.	made it difficult for you to do work (in or outside the home)?	**0**	**1**	**2**	**3**	**4**	**5**	**6**
2.	made it difficult for you to do activities outside of work (leisure activities)?	**0**	**1**	**2**	**3**	**4**	**5**	**6**
3.	made it difficult for you to spend time with friends and family members?	**0**	**1**	**2**	**3**	**4**	**5**	**6**
4.	affected your mood?	**0**	**1**	**2**	**3**	**4**	**5**	**6**
5.	affected your ability to do physical activities (like run, walk up stairs, play sports, do chores)?	**0**	**1**	**2**	**3**	**4**	**5**	**6**
6.	affected your sleep?	**0**	**1**	**2**	**3**	**4**	**5**	**6**

Adapted by the Neurobehavioral Group, NCI (8/23/13)
